# Refractory agitation in the NICU: challenges in prevention, diagnosis, and treatment

**DOI:** 10.3389/fped.2025.1504619

**Published:** 2025-02-27

**Authors:** Kim Beatty, Eunsung Cho, Jessica Biggs, Shawnee Daniel-McCalla, Johana Diaz

**Affiliations:** ^1^ Division of Neonatology, Department of Pediatrics, University of Maryland School of Medicine, Baltimore, MD, United States; ^2^ Department of Pharmacy, University of Maryland Medical Center, Baltimore, MD, United States

**Keywords:** ICU delirium, neonatal agitation, opiods, clonidine, benzodaizepine

## Abstract

In this paper we explore refractory agitation in the neonatal population, focusing on the limitations of existing evidence on appropriate prevention, diagnosis, and treatment options. We highlight seven patients identified in an urban single-center level IV NICU with agitation unresponsive to standard non-pharmacologic interventions and escalation of standard neurosedative medications. We analyzed baseline characteristics and clinical courses of these patients with the aim to identify the NICU subpopulation at greatest risk for development of refractory agitation and to gain insight into the potential benefits of alternative medical management of agitation on later neurodevelopment. Based on these experiences we propose a practical approach to infants at increased risk for refractory agitation including standardized screening guidelines and a clinical pathway for developmentally appropriate non-pharmacologic and pharmacologic management.

## 1 Introduction

Agitation is common in the Neonatal ICU (NICU) and often presents significant challenges in management. The underlying cause is multifactorial and further complicated by the disrupted neurodevelopment of a critically ill neonate.

Increased survival of extremely premature and low birth weight infants has led to prolonged hospitalizations and refractory agitation has become evident in critically ill and mechanically ventilated infants as they approach term-equivalent age (TEA). This is characterized by irritability and excessive psychomotor activity not responsive to non-pharmacologic management strategies and the need for multiple neurosedative medications at high doses.

In limited case-reports and case-series, this presentation has been identified as “neonatal delirium” based on comparisons to the adult and pediatric ICU populations as well as clinical response to atypical antipsychotics (1–8). While there is not sufficient evidence to elucidate if neonates experience delirium given their early stages of brain development, there is evidence to support that neonatal refractory agitation exists on the spectrum of ICU delirium. There is a lack of objective assessment tools to interpret their association. However, with the increasing numbers of extremely premature and low birth weight infants in the NICU, it is reasonable to anticipate a corresponding increase in the incidence of refractory agitation, making it imperative to develop a practical approach to managing these patients.

## 2 Methods

A single-center retrospective review of infants admitted to an urban level IV NICU who were administered an atypical antipsychotic. Medical records were searched for administration of quetiapine, risperidone, chlorpromazine, or haloperidol from January 2015 to July 2024. Included in our review was an additional patient with refractory agitation who was treated with phenobarbital due to pre-existing hypertriglyceridemia. Baseline characteristics, comorbidities, assessment tools, non-pharmacologic interventions, neurosedative initiation and dosing, antipsychotic use, and overall clinical response in these patients were analyzed using standard descriptive statistics. This review was Institutional Review Board exempt.

## 3 Results

Medical record search for administration of quetiapine, risperidone, chlorpromazine, or haloperidol from January 2015 to July 2024 identified 6 patients. An additional patient with refractory agitation who was treated with phenobarbital due to pre-existing hypertriglyceridemia. In total, seven patients met inclusion criteria. Patient characteristics, comorbidities, neurosedative and antipsychotic use are summarized in Table 1. The mean gestational age was 25 weeks (range 23.2–28.5) with a mean birth weight of 608 grams (range 485–750). The average length of hospitalization was 208 days (range 136–301). One patient was discharged home directly from the level IV NICU, five required transfer to a rehabilitation facility or other institution, and one died during initial hospitalization related to pulmonary hypertensive crisis.

**Table 1:** Patient characteristics.

Patient	1	2	3	4	5	6	7	Mean
GA	23w6d	28w5d	25w4d	25w2d	24w0d	23w3d	23w2d	24w6d
BW (g)	485	750	645	600	655	540	580	608
Race	White	White	Black	Black	Black	Black	Hispanic	–
Duration of hospitalization (days)	301	159	259	136	143	229	201	208
Disposition	Home	Deceased	Transfer	Transfer	Transfer	Transfer	Transfer	–
Steroid courses	4	1	5	2	5	3	2	3
BPD severity	Severe	Severe	Severe	Severe	Severe	Severe	Severe	–
Echo findings	PDA, PFO, dysplastic pulm valve	PDA, VSD, ASD	PDA	ASD, mod outlet VSD	PDA, PFO	RA/IVC thrombus, PDA	PDA, ASD vs. PFO	–
MRI findings	Left thalamus infarct	Sequelae of prior extra-axial hemorrhage, encephalomalacia	Cerebral volume loss, thinning of corpus callosum	Not performed	Moderate ventriculomegaly	Absent septum pellucidum, mild ventriculomegaly	Not performed	-
Antibiotic courses	9	5	10	9	6	9	13	8.7
Surgeries	GT, laser	GT, inguinal hernia repair	GT, trach	GJ, trach, VSD repair	GT, trach, circ	Bowel resection, ostomy closure, GT, trach	Bowel resection, ileostomy, GT, trach, laser	–
PMA when continuous opioid started	26w3d	41w2d	34w0d	38w6d	31w5d	28w2d	27w3d	–
Duration of continuous opioid (days)	166	45	216	147	87	125	173	137
PMA when continuous benzodiazepine started	44w2d	41w3d	40w3d	40w3d	35w1d	59w5	28w1d	–
Duration of continuous benzodiazepine (days)	91	35	130	95	105	47	72	82
PMA when atypical antipsychotic started	52w6d	46w2d	53w2d	49w2d	38w6d	52w2d	36w4d^a^	–
Duration of atypical antipsychotic (days)	117	33	80	65	32	39	109	68
Adverse effects of antipsychotic therapy	None	Elevated triglycerides	None	None	None	None	None	–

^a^ Phenobarbital used due to preexisting hypertriglyceridemia.

All patients required intubation on the first day of life with high frequency ventilation. All had severe BPD and received at least one postnatal steroid course for BPD (mean 3, range 1–5). Five (71%) underwent tracheostomy placement. All developed BPD-associated pulmonary hypertension and had at least one episode of pulmonary hypertensive crisis requiring inhaled nitric oxide and neuromuscular blockade. 6 (86%) needed long term treatment with sildenafil. 6 (86%) had PDA, 2 of which (29%) were treated with ibuprofen or acetaminophen. Additional significant echocardiogram findings included dysplastic pulmonary valve (14%), VSD (29%), ASD (43%), and one patient with a central venous catheter-related intracardiac thrombus. 6 (86%) had significant feeding intolerance, 3 (43%) had at least one episode of medical NEC, and all 7 (100%) underwent gastrostomy or gastrojejunostomy tube placement. All had multiple infections and antibiotic courses (mean 8.4). Only 2 (29%) had significant intracranial hemorrhages. Of the five patients who had brain MRI, none had evidence of PVL or significant hydrocephalus. Two patients had developmental evaluations after discharge (CAT/CLAMS), and both were found to have global developmental delays.

All patients were exposed to multiple neurosedative medications including prolonged continuous infusions of opiates and benzodiazepines. PMA (Postmenstrual age) at time of opiate exposure ranged from 28 to 41 weeks with an average duration of 137 days (range 45–216) for continuous infusions and ranged from 28 to 49 weeks with an average duration of 82 days (range 35–130 days) for benzodiazepine continuous infusions.

All patients were TEA when an atypical antipsychotic was started (range 38.6–52.6). 5 (71%) remained on mechanical ventilation and all were on at least two continuous infusions of neurosedative medications. 6 patients (86%) initially received quetiapine; 5 had decreased agitation scores (N-PASS, WAT, CAPD), decreased number of PRN medication doses, and decreased respiratory support. One developed hypertriglyceridemia and was switched to risperidone, with a similar response. Another patient was transitioned to chlorpromazine from quetiapine due to no changes in CAPD scores. The final patient was started on phenobarbital due to his pre-existing hyper-triglyceridemia. Average duration of treatment with antipsychotics or phenobarbital was 68 days (range 32–117 days); antipsychotics were discontinued once patients were weaned off IV sedation.

## 4 Proposed clinical pathway

**Figure 1:**
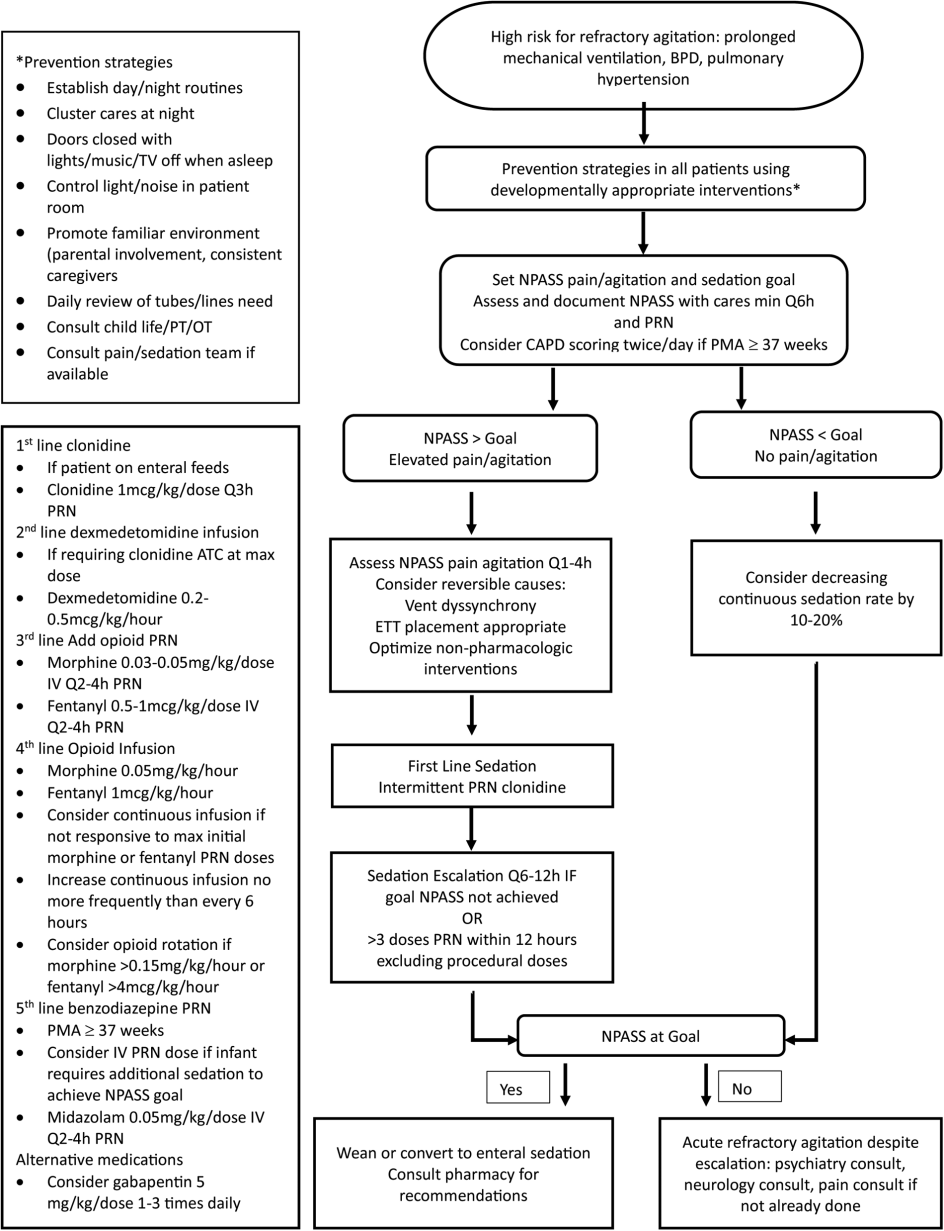
Refractory agitation in the NICU: clinical management pathway.

Figure 1 outlines the proposed clinical pathway for non-pharmacologic and pharmacologic management of NICU patients at risk for the development of refractory agitation. Management begins with developmentally appropriate prevention strategies. Monitoring using NPASS pain/agitation and sedation scoring. Once TEA, CAPD scoring in combination with NPASS should be considered. Goal scores should be individualized to account for baseline patient behaviors and may need to be adjusted over time. If scores remain elevated after reversible causes have been addressed and non-pharmacologic interventions have been optimized, pharmacologic sedation may be necessary.

## 5 Discussion

Recognition of refractory agitation in the NICU is increasing. Variations in terminology to describe this phenomenon, limited understanding of its pathophysiology, and lack of defined diagnostic criteria have made it difficult to determine risk factors and incidence. There is conflicting evidence on available scoring tools and uncertainty regarding pharmacologic treatment strategies with little evidence for the long-term safety and efficacy. Through this review we aim to define refractory agitation and its association with ICU delirium, identify risk factors, and present a guideline for prevention and management.

### 5.1 What is refractory agitation and how does it relate to delirium?

Delirium is a disturbance in attention and cognition from baseline that develops over a short period of time, tends to fluctuate in severity, and is not explained by another neurocognitive disorder (9). In neonates, identifying disturbances in cognition is challenging due to their changing cognitive baseline and high risk for other neurodevelopmental disturbances related to prematurity and complex comorbidities. It is unclear if premature infants have the level of brain development to experience the neurobiological processes thought to underly the pathogenesis of delirium (10). Despite these challenges, there are several factors to suggest that a pathology on the spectrum of delirium can occur in NICU patients.

The pathogenesis is not fully understood; several hypotheses have been proposed, all resulting in dysregulation of neuronal activity including neurotransmitter imbalance, oxidative stress with subsequent free radical-mediated injury, and neuroinflammation leading to elevated glucocorticoids and increased cytokine expression (1, 4). It is possible that there is increased susceptibility to delirium in neonates due to their developing nervous system and vulnerability to inflammatory conditions and oxygen toxicity; the lack of standardized diagnostic criteria and rigorously validated assessment tools for this unique population prevent accurate recognition.

Recognized delirium risk factors in adult and pediatric patients include prolonged hospitalization, baseline cognitive dysfunction, need for invasive mechanical ventilation, suboptimal pain or agitation management, and exposure to corticosteroids and neurosedative medications, all of which are pervasive in the NICU population (1, 3, 5).

Most cases of refractory agitation in the NICU are reported to occur at or around TEA, as seen in our patient population. By this age, patients should have a determined cognitive and behavioral baseline in which a change can be recognized. Although limited to case-reports and case-series they have demonstrated response to conventional delirium treatment with atypical antipsychotics (2, 6–8).

### 5.2 Risk factors for refractory agitation

Based on the characteristics identified in our study population and existing literature, several risk factors for the development of refractory agitation were identified. These include extreme prematurity, ELBW status, severe bronchopulmonary dysplasia [BPD] with the need for prolonged mechanical ventilation, multiple steroid courses, pulmonary hypertension, recurrent infections, and feeding intolerance. Interestingly, severe intracranial hemorrhage or PVL did not appear to be a significant risk factor.

Pulmonary hypertension is the most significant risk factor. BPD is a major complication of prematurity with increasing incidence due to the improved survival of the most premature neonates. About 25% of infants with moderate to severe BPD will develop secondary pulmonary hypertension (11). Continuous infusions of multiple neurosedative medications are often required in the management of pulmonary hypertension (12). Even after resolution of PH crisis, weaning off these medications presents a challenge and can predispose infants to repeat PH crises. While the risk factors are largely non-modifiable, recognizing these features can help identify those patients at greatest risk allowing for earlier recognition and intervention.

### 5.3 Recognition: limitations of scoring tools

One challenge in early recognition of refractory agitation is the lack of validated diagnostic tools. Available tools are based on subjective evaluation of behavioral responses and were primarily developed to assess acute responses to procedural pain or stress from handling. While there are several assessment tools available, our NICU uses the Neonatal Pain, Agitation and Sedation Scale (N-PASS) and Cornell Assessment of Pediatric Delirium (CAPD) scale to evaluate patients at increased risk for refractory agitation.

The N-PASS utilizes 5 domains: crying/irritability, behavior/state, facial expression, extremities/tone, and vital signs to assess pain/agitation and sedation with an assigned score between 0 and 10 for pain/agitation and 0 to −10 for sedation with additional points assigned based on corrected gestational age (13). It has been validated for evaluating pain and agitation in infants born as early as 23 weeks gestation, mechanically ventilated patients, and in cases of prolonged pain and agitation (13–15). The impact of neurologic abnormalities on validity and reliability of the N-PASS, however, have not been clearly determined (13). Additionally, it can be difficult to differentiate pain and agitation in neonates as they present with similar behavioral and physiologic manifestations. For these reasons, specific target N-PASS scores may need to be adjusted for individual patients based on their own baseline and interpreted within the context of their clinical status.

The CAPD is an observation tool which evaluates eye contact, purposeful actions, awareness of surroundings, communication, restlessness, inconsolability, activity level in awake states, and timing and response to interactions. It has been validated as a screening tool for delirium in the pediatric ICU, including infants and newborns (validation included only 25 infants, with 7 < 1 month of age) (16). Despite its increasing use, with an estimated 22% of TEA infants in the NICU screen positive for delirium, the CAPD has not been validated in the NICU setting (3). The impact of prematurity, neurodevelopmental impairment, and invasive ventilation on the predictive value of these scores is not well understood. Higher rates of false positive screens have been demonstrated in each of these groups (16, 17). While developmental anchor points can be used to improve accuracy of screening in children under 2, they are difficult to apply in the NICU given the atypical neurodevelopmental trajectories.

Kaur et al (18), recognizing the challenges of using CAPD scores in patients with developmental delay, demonstrated that a combined CAPD score >9 with a fluctuation in Richmond agitation sedation scale score of >2 was both sensitive and specific for detecting delirium in developmentally delayed PICU patients. Despite their individual limitations, using the N-PASS and CAPD scores together and considering each patient's individual cognitive and behavioral baseline can help improve the usefulness of these tools in identifying refractory agitation and response to treatment. It is important to note that these are screening tools and cannot be used to diagnose refractory agitation or delirium.

### 5.4 Pharmacologic treatments

Management of pain and agitation remains a complex challenge in the NICU. The known negative neurodevelopmental outcomes of untreated pain and stress must be weighed against the detrimental effects of opioids and benzodiazepines and the relatively unknown long-term safety and efficacy of emerging therapies including alpha-2 receptor agonists and atypical antipsychotics (19, 20). These challenges are magnified in the extremely premature neonate who will likely require prolonged exposure to any number of these drugs.

Analgesics and sedatives are used frequently in the NICU to manage pain and agitation, promote comfort and safety (21). Administration of opioids and benzodiazepines has been associated with altered brain development and poor neurodevelopmental outcomes ^(^19, 22). Prolonged use (>7 days) of opioids and benzodiazepines has been associated with lower Bayley scales of infant development (BSID-III) cognitive, motor, and language scores for premature infants examined at 2 years (23). It is also associated with analgesic and sedative tolerance and physical dependence, necessitating escalating doses and/or addition of adjunctive agents to achieve adequate pain and sedation control (24).

Antipsychotics have become a therapy of interest for the management of refractory agitation and delirium. Antipsychotics regulate neurotransmitter levels essential in affecting mood and behavior. First-generation, or typical, antipsychotics (haloperidol, chlorpromazine) are dopamine receptor antagonists. Second-generation, or atypical, antipsychotics (quetiapine, risperidone) are dopamine and serotonin receptor antagonists. By blocking these receptors, antipsychotics decrease the rate of elimination of these neurotransmitters released by electrical stimulation of neurons (25). Although antipsychotics have not been FDA approved for pharmacological delirium treatment in pediatric patients and the PANDEM guidelines recommend that antipsychotics should not be routinely used to prevent or decrease the duration of delirium in a critically ill patient. PANDEM guidelines do recommend consideration of antipsychotics for the management of severe delirium manifestations with caution of possible adverse drug effects (26). Further, there is no data available on long term neurodevelopmental impacts of these medications.

Quetiapine is an oral atypical antipsychotic that has been demonstrated to be tolerated in pediatric ICU patients as young as 2 months of age (27, 28). Common adverse events associated with quetiapine include dyslipidemia, extrapyramidal symptoms, hyperglycemia, temperature dysregulation, sedation, weight gain and QT prolongation. Other less common adverse effects include neuroleptic malignant syndrome, hypothyroidism, and hematologic abnormalities. Anticholinergic effects including constipation, urinary retention, xerostomia, and blurred vision are also possible although quetiapine's anticholinergic activity is low in comparison to other second-generation antipsychotics. Quetiapine results in symptom improvement within 24–72 h after initiation (29, 30).

Risperidone is an atypical antipsychotic that is formulated in injectable (intramuscular, subcutaneous) and enteral formulations. It is commercially available in various tablet strengths as well as a 1 mg/ml oral solution, which allows for more precise tailoring and titration of pediatric dosing as well as easier administration in infant or pediatric patients unable to swallow tablets. Common side effects include somnolence, fatigue, hyperprolactinemia, weight gain, constipation, nausea, vomiting, dizziness, and extrapyramidal side effects (25, 31, 32). Given its atypical antipsychotic status, extrapyramidal side effects should theoretically be less than a typical antipsychotic agent. When compared with quetiapine, risperidone has been associated with more extrapyramidal symptoms (33). Data regarding dosing is limited in the infant and younger pediatric populations. Symptom improvement may be seen within 24 h of starting risperidone with resolution of delirium within 3.5–12 days (34).

Alpha-2 agonists, specifically dexmedetomidine and clonidine, provide analgesia, anxiolysis, and sedation without impacting respiratory drive or gastrointestinal motility (19). Pre-synaptic activation of alpha-2 adrenergic receptors in the central nervous system leads to the inhibition of norepinephrine release causing termination of pain signals. These medications also stimulate the release of substance P leading to analgesia, potentiating the effects of opioids (35–37). Clonidine is available as an oral liquid, tablet, or transdermal patch with an onset of action of 30–60 min (36, 37). Dexmedetomidine is administered as an IV infusion with an onset of action of 5–10 min and is 8 times more selective for alpha-2 adrenergic receptors than clonidine (38). Although limited, there is data to suggest that the use of alpha-2 agonists may be effective in reducing the need for adjunctive sedation or analgesia, decreasing the duration of mechanical ventilation, and accelerating the attainment of full enteral feeds (39). Additionally, the use of dexmedetomidine has been associated with a decrease in overall opioid and benzodiazepine exposure (40, 41). Post-synaptic activation of alpha-2 receptors inhibits symptomatic activity leading to decreases in blood pressure and heart rate (33).

Other medications such as gabapentin, with a better-known safety profile, could be considered. Gabapentin, a gamma-aminobutyric acid analog, is increasingly used in infants with neuro-irritability of various etiologies (42). Its use is associated with decreased N-PASS scores and reduced need for analgesic or sedative medications, though data regarding dosing, efficacy and long-term neurodevelopmental impact remains limited (22, 42, 43).

### 5.5 Proposed clinical pathway

Our proposed clinical pathway focuses on timely intervention and limiting exposure to opioids and benzodiazepines by prioritizing non-pharmacologic interventions and alpha-2 agonist medications.

Several non-pharmacologic interventions have been shown to reduce physiologic and behavioral stress responses. The efficacy of these interventions is dependent upon neonatal maturity and must be tailored based on PMA (44–47). They should be routinely incorporated into the care of all patients admitted to the NICU to prevent stress and agitation. Standard measures should include multidisciplinary involvement with establishment of daily routines, controlling light and sound exposure, encouraging parental involvement, creating a familiar environment, promoting uninterrupted sleep, clustering patient care activities, and minimizing painful procedures.

If pharmacological intervention is needed, intermittent dosing should be trialed before escalating to continuous infusions and alpha-2 agonists should be used first-line, when able. Opioids and benzodiazepines may be necessary; however, their use should be limited based on their known detrimental neurodevelopmental effects. Should agitation persist despite the above interventions, there is not sufficient evidence to routinely recommend the use of atypical antipsychotics in the neonatal population. Consultation with pediatric psychiatry, neurology, and pain team, if available, is recommended prior to initiation of medications outside of the clinical pathway.

### 5.6 Conclusion

Although further understanding of the underlying etiology of refractory agitation, its potential association with delirium, and dedicated research on the safety and neurodevelopmental impacts of emerging treatment strategies is critical, we must begin with recognition of this phenomenon in our unique patient population. Identifying high-risk patients can enable the use of non-pharmacologic neuroprotective measures that may prevent refractory agitation. Improved surveillance of this high-risk population may allow for earlier intervention to minimize symptoms.

We acknowledge that this pathway is limited by the availability of existing data, but necessary given the pervasiveness of agitation in the neonatal population and its potential long-term detrimental effects. We aim to call attention to the need for further research into the efficacy of current management strategies for and long-term impacts of neonatal agitation.

## Data Availability

The data analyzed in this study is subject to the following licenses/restrictions: Requests to access these datasets should be directed to kbeatty@som.umaryland.edu, jdiaz@som.umaryland.edu.
